# Control of Anisotropy and Magnetic Hyperthermia Effect by Addition of Cobalt on Magnetite Nanoparticles

**DOI:** 10.1021/acsami.4c03343

**Published:** 2024-07-15

**Authors:** Adriele Aparecida de Almeida, Fernando Fabris, Gustavo Soares da Silva, Kleber Roberto Pirota, Marcelo Knobel, Diego Muraca

**Affiliations:** Instituto de Física “Gleb Wataghin” - Universidade de Campinas, 13083-859 Campinas, São Paulo, Brazil

**Keywords:** Magnetic hyperthermia, Nanoparticles, Specific power absorption, Magnetic anisotropy, Néel relaxation

## Abstract

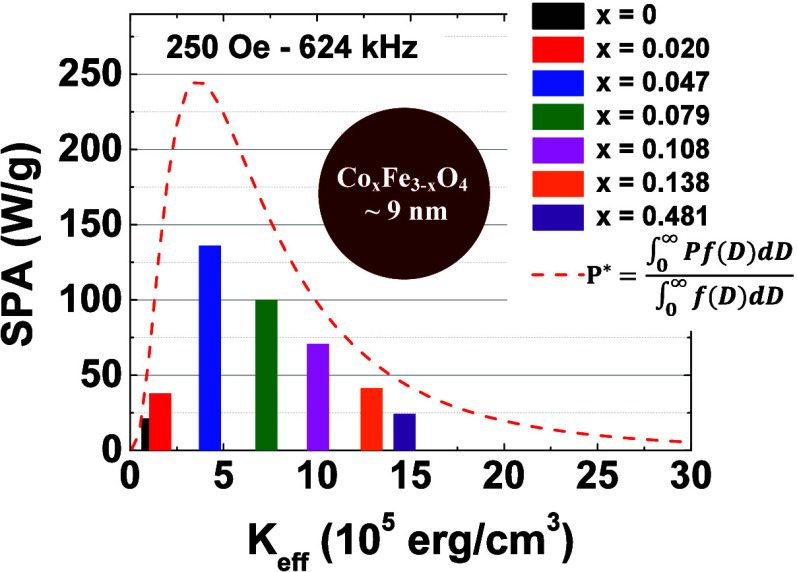

Magnetic hyperthermia (MH) has emerged as a promising technology with diverse applications in medical and technological fields, leveraging the remote induction of temperature elevation through an alternating magnetic field. While Fe_3_O_4_ nanoparticles with an average size around 12–25 nm are commonly employed in MH systems, this study introduces a strategy to produce smaller particles (less than or equal to 10 nm) with enhanced heating efficiency, as measured by specific power absorption (SPA). We conducted an exhaustive and detailed investigation into the morphological and magnetic properties of Co_*x*_Fe_3–*x*_O_4_ nanoparticles, aiming to optimize their MH response. By varying the Co content, we successfully tuned the effective magnetic anisotropy while maintaining saturation magnetization nearly constant. The MH analysis indicates that these nanoparticles predominantly heat through the Néel mechanism, demonstrating robust reproducibility across different concentrations, viscosity mediums, and ac field conditions. Notably, we identified an optimal anisotropy or Co concentration that maximizes SPA, crucial for developing magnetic systems requiring particles with specific sizes. This work contributes to advancing the understanding and application of MH, particularly in tailoring nanoparticle properties for targeted and efficient heat generation in various contexts.

## Introduction

Magnetic hyperthermia (MH) is a technique that involves raising the temperature of a medium by exploiting the magnetic losses exhibited by magnetic nanoparticles when subjected to an alternating current (ac) magnetic field at radiofrequency levels. In this process, the targeted medium, often biological tissues or fluids^1−5^ or viscous fluids,^6−8^ experiences controlled heating due to the energy dissipation from the magnetic materials. The choice of magnetic nanoparticles provides versatility, and the application of an ac magnetic field in the radiofrequency range allows precise control over the induced heat,^5,9,10^ making MH a promising approach in various fields, particularly in targeted medical treatments where controlled and localized heating is advantageous^1−3,5^ and, too, to control the viscosity of the petroleum where changing the rheological properties of the petroleum can facilitate its transport.^7,8^ Ferrite nanoparticles serve as compelling systems for MH due to the ability to manipulate their magnetic properties through composition and size control.^11,12^ Beyond this tunability, they exhibit a plethora of potential applications, adding to their overall significance in various fields.^13^ It is important to note that while the hydrophobic nature of the nanoparticles used in this study may limit their direct suitability for biomedical applications, it is worth noting that surface modifications of nanoparticles can be implemented to render these nanoparticles suitable for biomedical uses. It is worth noting that nanoparticles with hydrophobic surfaces exhibit a strong affinity for oil, making them essential for controlling petroleum viscosity, for example. These facts highlight possible versatility across different industrial and medical applications.

In MH of monodomain magnetic nanoparticles (MNPs) within a viscous medium, two main relaxation mechanisms are important: the viscous mechanism (Brown) and the moment inversion mechanism against an energy barrier (Néel).^14^ That is, mechanical or Brown mechanism (*τ*_B_) relies on the rheological properties of the system that is given by
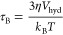
1where η is the viscosity of the medium, *V*_hyd_ the hydro dynamic volume of the MNPs, and *k*_B_*T* the thermal energy. In contrast, Néel relaxation (*τ*_N_) relies on the magnetic properties of the particles and is expressed by
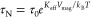
2where *K*_eff_ is the effective magnetic anisotropy, *V*_mag_ the magnetic volume of the single-domain MNPs, and τ_0_ is the characteristic relaxation time (typically between 10^–9^–10^–11^ s).^14,15^ It is known that the dominating relaxation process will be the one with the shorter relaxation time, where the effective relaxation time (τ) is given by^15^. The predominance of one mechanism over the other is associated with different intrinsic properties of the particles, such as *K*_eff_ and *V*_mag_, as well as the viscosity of the medium (η) in which they are dispersed, and the *V*_hyd_ in this medium.^15,16^ Furthermore, the heat generation efficiency of MNPs in a hyperthermia experiment, referred to as specific power absorption (SPA), will depend on the parameters of the alternating magnetic field, such as frequency (*f*) and amplitude (*H*_0_).^15^ A nuanced understanding of these factors is essential for a comprehensive grasp of hyperthermia dynamics, offering valuable insights for optimizing its applications.

The key parameter in MH is the SPA of the MNPs and is determined by the magnetic, morphological, and rheological properties of the solvent/MNPs.^15^ The SPA is frequently utilized to assess the effectiveness of a MH system in generating heat in target regions.

To maintain experiment reproducibility is important that MNPs intended for hyperthermia applications should be fabricated in a way that favors the dominance of *τ*_N_,^1,6,12,17^ as heating efficiency relies solely on the intrinsic magnetic properties of MNPs and remains independent of the variable viscosity in different medium. Additionally, MNPs tend to aggregate when dispersed in some medium.^6,18,19^ Under these conditions, the Néel mechanism undergoes changes due to interactions, allowing for the adjustment of magnetics properties to maintain high heating efficiency. Meanwhile, as we saw in the equation above, the Brown relaxation time significantly and inevitably increases, leading to the loss of absorption capability within a reasonable frequency range for efficient hyperthermia application.

The thermal power or power dissipation (*P*) generated by MNPs under an alternating field, also measured in terms of SPA, can be analytically described by^15^
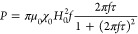
3where μ_0_ is the permeability in a vacuum. χ_0_ is the equilibrium susceptibility defined in the low-field region as , where ρ is density and *M*_s_ the saturation magnetization of the material.^18,20^ This is then an analytical form to calculate the power dissipated by superparamagnetic particles subjected to an external field within the limits of the linear response theory (LRT).^15,17^

Briefly, the thermal power generated by MNPs in the presence of an alternating magnetic field depends on field parameters, specifically its power, and the dissipation related to the ratio between the field frequency and the magnetic moment relaxation time. Therefore, the heat generated by magnetic losses is directly linked to the relaxation mechanisms of the magnetic moment, *τ*_B_ and/or *τ*_N_, transforming electromagnetic energy into thermal energy.

In a hyperthermia experiment, the SPA is related to the heating rate through a quick thermodynamic analysis, assuming some factors: first, there are no thermal losses, especially to the surroundings, and second, the mass of MNPs dispersed in the medium is much smaller than the medium. Therefore, it can be expressed as
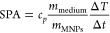
4where *c*_*p*_ is the specific heat of the medium, *m*_medium_ is the mass of the medium, *m*_MNPs_ is the mass of MNPs, and  is the heating rate obtained from the initial slope of the heating curve.

**Figure 1:**
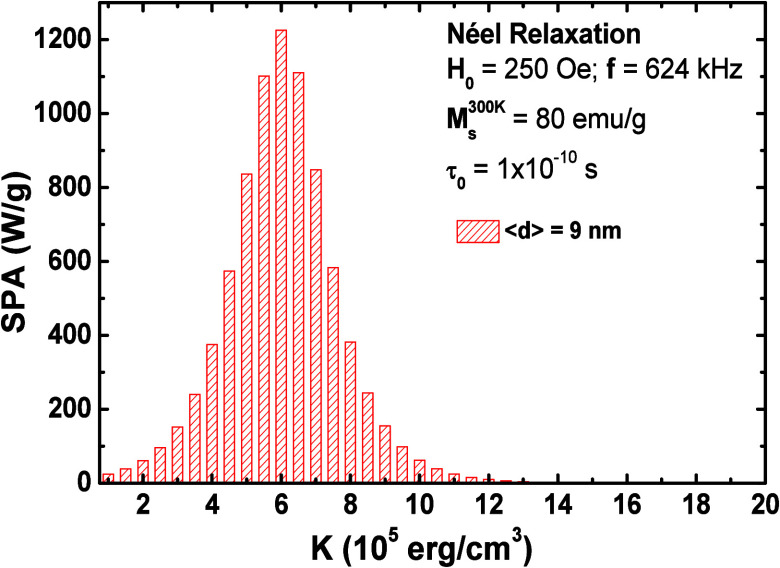
Estimated power dissipation as a function of magnetic anisotropy obtained using eq 3 and considering exclusively the Néel relaxation time. The magnetic parameters used in the equation are cited in the graph.

Among the various types of MNPs explored for MH, magnetite nanoparticles (Fe_3_O_4_) emerge as the most extensively investigated due to their favorable magnetic properties, biocompatibility, and cost-effectiveness. However, considering only the Néel relaxation time, an additional complexity arises in optimizing the performance of MNPs. For applications requiring MNPs with a diameter less than or equal to ∼10 nm, a reduction in Fe_3_O_4_ efficiency for MH is observed,^21,22^ with an ideal size around 12–25 nm.^21,22^ As evidenced in eq 3, considering only the Néel relaxation, adjusting the size of MNPs to achieve an ideal SPA is more challenging than tuning *K*_eff_. Given this, we utilized eq 3, for a fixed size of 9 nm (∼ average size among the 7 samples this work), and varying *K*_eff_ from 1 × 10^5^ erg/cm^3^ (Fe_3_O_4_ anisotropy)^23,24^ to 20 × 10^5^ erg/cm^3^ (Co_1_Fe_2_O_4_ anisotropy)^24−27^ we can calculate the expected *P* values, as depicted in the graph of Figure 1. We observed that to achieve optimal efficiency in MH with MNPs of diameter less than 10 nm, heating through the Néel mechanism, it is crucial to increase the anisotropy of Fe_3_O_4_.

Previous studies have explored doping magnetite with Co to modulate heat absorption in magnetic hyperthermia.^28−30^ However, the coprecipitation synthesis method used resulted in increased nanoparticle size and decreased magnetic saturation with higher cobalt content,^29,30^ limiting precise control over size and magnetic anisotropy. Conversely, Fantechi et al.^28^ synthesized Co_*x*_Fe_3–*x*_O_4_ particles via thermal decomposition of acetylacetonates, achieving an average size of around 8 nm with varying *x* from 0 to 1. They reported an unexpected reduction in magnetic anisotropy for *x* > 0.6 and maximum heat absorption at *x* = 0.6.

In this study, we delved into the impact of varying the Co content on the MH properties of cobalt ferrite Co_*x*_Fe_3–*x*_O_4_ type nanoparticles. As the value of *x* increased from 0 to 0.481 in particles with an average size of around 9 nm, we observed a continuously increase in *K*_eff_. MH assessments conducted in two distinct media, one with low^31^ and the other with high^6,32^ viscosity (η), revealed similar SPA values, indicating that MNPs predominantly undergo heating through the Néel relaxation mechanism. Notably, the SPA values exhibited a clear dependence on *K*_eff_, allowing us to pinpoint an optimal SPA for a specific particle size with *x* = 0.047.

## Materials and Methods

Co_*x*_Fe_3–*x*_O_4_ oleic acid-coated MNPs reported here were obtained by high temperature thermal decomposition of the Fe(III) and Co(II) acetylacetonates (6 mMol totals/together) dispersed in 120 mMol of benzyl ether solvent, 6 mMol of 1,2-octanediol, oleic acid (9 mMol), and oleylamine (9 mMol) (surfactants). Stoichiometry of each sample was controlled by the acetylacetonates ratio used in the solution from where different compositions were obtained (see Table 1). For all samples, the solution was heated between ∼100–120 °C during 20 min under N_2_ flow and intense mechanical stirring. After that, N_2_ was removed, and the solution was slowly heated until the reflux condition (298 °C) with a heating rate of 3 °C/min. The solution was kept in reflux during 60 min.

**Table 1:** Chemistry and Structural Results Obtained for All Co_*x*_Fe_3–*x*_O_4_ Samples^a^

Co_*x*_Fe_3–*x*_O_4_sample name	Nominal stoichiometry	TXRF stoichiometry	⟨*d*⟩_XRD_ (nm)	⟨*d*⟩_TEM_ (nm)	σ_TEM_
*x* = 0	Fe_3_O_4_	Fe_3_O_4_	9.7	7.9	0.2
*x* = 0.020	Co_0.02_Fe_2.98_O_4_	Co_0.020_Fe_2.980_O_4_	8.5	8.4	0.2
*x* = 0.047	Co_0.04_Fe_2.96_O_4_	Co_0.047_Fe_2.953_O_4_	12	10.6	0.4
*x* = 0.079	Co_0.06_Fe_2.94_O_4_	Co_0.079_Fe_2.921_O_4_	11	9.4	0.3
*x* = 0.108	Co_0.08_Fe_2.92_O_4_	Co_0.108_Fe_2.892_O_4_	9.9	8.1	0.3
*x* = 0.134	Co_0.1_Fe_2.9_O_4_	Co_0.134_Fe_2.866_O_4_	9.5	8.7	0.3
*x* = 0.481	Co_0.5_Fe_2.5_O_4_	Co_0.481_Fe_2.519_O_4_	9.1	9.1	0.2

a Given sample names, nominal stoichiometry, the stoichiometry obtained by TXRF analysis, average diameter ⟨*d*⟩_XRD_ obtained from XRD pattern, and average diameter ⟨*d*⟩_TEM_ and size dispersion (σ_TEM_) obtained from the TEM images analyzes.

The chemical composition of the samples was determined by total reflection X-ray fluorescence (TXRF) measurements, with a S4 T-STAR Bruker equipped with a Mo X-ray source. In the TXRF procedure, powdered samples were adhered to sample holders using a drop of each sample dispersed in a mixture of hexane and water. Measurements were carried out postevaporation of hexane and water. Structural characterization was carried out using a X-ray diffraction (XRD) measurements with D2 Phaser diffractometer (Bruker) using Cu Kα radiation (λ = 1.5418 A°), operating at 30 kV and 10 mA, in the 2θ range from 25° to 90° with steps of 0.02° at room-temperature. Samples were cleaned with acetone to remove organic material, and the powder was deposited in the sample holder. The size, diameter dispersion, and high-resolution (HRTEM) analysis of our MNPs were conducted using transmission electron microscopy (TEM) on a JEOL JEM 2100F, operating at 200 kV. For the microscopy experiment, samples were dispersed in toluene and then prepared by drying a drop of the suspension on a Ted Pella ultrathin cooper film on a holey carbon. The diameter histogram was generated by measuring the diameter of at least ∼400 MNPs, and the average diameter (⟨*d*⟩) and dispersion (σ) were determined by fitting the data with a log-normal distribution.

Percentage of organic compound in the nanoparticles to have accuracy in mass to normalize magnetization experimental data with the oxide amount was determined for each sample by means of differential thermal thermogravimetric analyzer (TGA), model 2950 TGA HR V5.3C. Samples powder was heating up to 1000 °C with a heating rate of 5 °C/min in Ar flux (60 mL/min) while weight was measured. This analysis revealed that approximately 10–15% of the total mass in all samples consisted of organic compounds. This fraction of organic material in the MNPs was subsequently subtracted from the overall sample mass during magnetic measurements. The magnetic characterization needed in order to understand the MNPs response in MH experiments are the *M*_*s*_, the *K*_eff_ constant, and blocking temperature (*T*_B_). *M*_*s*_ and *K*_eff_ were determined by magnetization measurements as a function of the applied field (*M*(*H*)) measured at various temperatures. Blocking temperature distribution (*f*(*T*_B_)) of each sample was obtained from the magnetization curves as a function of temperature (*M*(*T*)) measured in the zero-field-cooling and field-cooling modes (ZFC and FC curves, respectively) by^6^. The average value of the *T*_B_ (⟨*T*_B_⟩) was calculated from the Kloster et al. reference.^33^ These magnetic measurements were performed in a commercial SQUID magnetometer (Quantum Design, MPMS3), and the samples were conditioned by dispersing the MNPs in epoxy resin (∼0.03 wt %), avoiding the agglomeration and physical rotation of the nanoparticles.

For the magnetic hyperthermia experiments, the MNPs were dispersed in paraffin and toluene at a controlled concentration and performed in two different commercial models (nB nanoScale Biomagnetics company D5 Series model from Spain and Fives company Celes MP 6 kW from France), with a working amplitude of 200–600 Oe and frequency of 250–624 kHz.

To avoid any effects of dipolar interactions all magnetic measures as well as specific absorption rate or calorimetric properties were done in a very low nanoparticles concentration, guaranteeing a strong dispersion of them.

## Results and Discussion

In order to tune magnetic properties of the Co_*x*_Fe_3–*x*_O_4_ samples, the chemical composition of obtained MNPs is of great importance, since the magneto-hyperthermia properties strongly determined them. To enhance SPA in nanoparticles with a size around 9 nm, the ratio of Fe and Co in the ferrite nanoparticles was altered, changing the magnitude of the internal magnetic moment due to the presence of the ion Co. But in the composition range we are working, the most significant effect should be changes on effective anisotropy constant of the system, *K*_eff_, and to a lesser degree on saturation magnetization, *M*_s_, as we will see in the magnetic results below.

The effective stoichiometry of each resulting sample was determined using TXRF (presented in Table 1). From the table, we see that the amount of Co in the final stoichiometry of the MNPs is not the same molar ratio expected from the amount of the precursors used in the synthesis; however, it is very close to what was expected.

**Figure 2:**
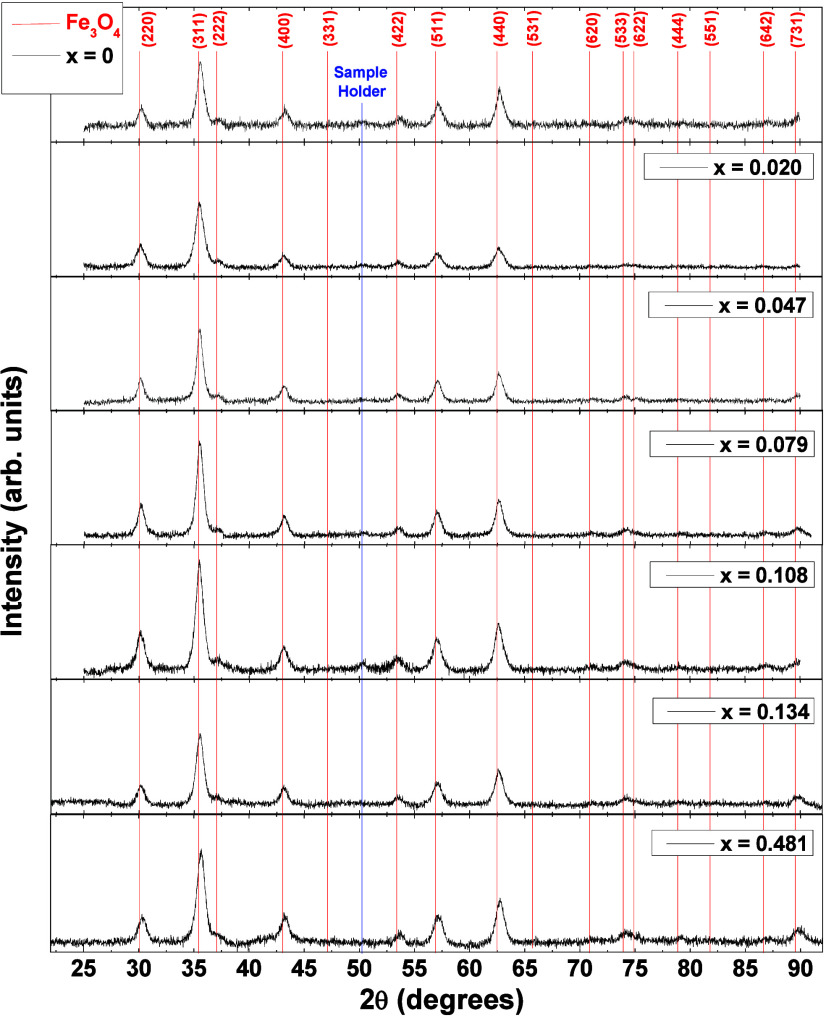
XRD pattern of the Co_*x*_Fe_3–*x*_O_4_ samples. All peaks are indexed with the magnetite where the red bars correspond to the diffraction peaks according to the COD number 1010369 (Fe_3_O_4_). The blue bar, in ∼50.1°, corresponds to a peak from the glass sample holder.

Room temperature XRD of all the samples synthesized, with different cobalt concentrations, are shown in Figure 2. The crystalline phase of the samples contains strong diffraction peaks at 2θ = 30.2°, 35.5°, 43.2°, 53.5°, 57.1°, and 62.8°, corresponding to (220), (311), (400), (422), (511), and (440) crystalline planes of cubic magnetite phase, respectively (COD number 1010369). The average crystallite diameter  was calculated according to the Scherrer’s equation, where it was considered as 0.9 shape parameter in the Scherrer equation,^34^ λ is the wavelength of the X-ray (Cu Kα, λ = 1.5418 *A*°), β is the full-width at half-maximum (fwhm) of the diffraction peak studied, and θ_XRD_ the Bragg angle. With a pseudo-Voigt fit (linear combination of the Gaussian and Lorentzian function) on the peak [113] of Figure 2, the value of the fwhm is obtained and, consequently, an average crystallite size for all samples and is found in Table 1. Using the Scherrer’s equation is very close to the data of TEM images.

**Figure 3:**
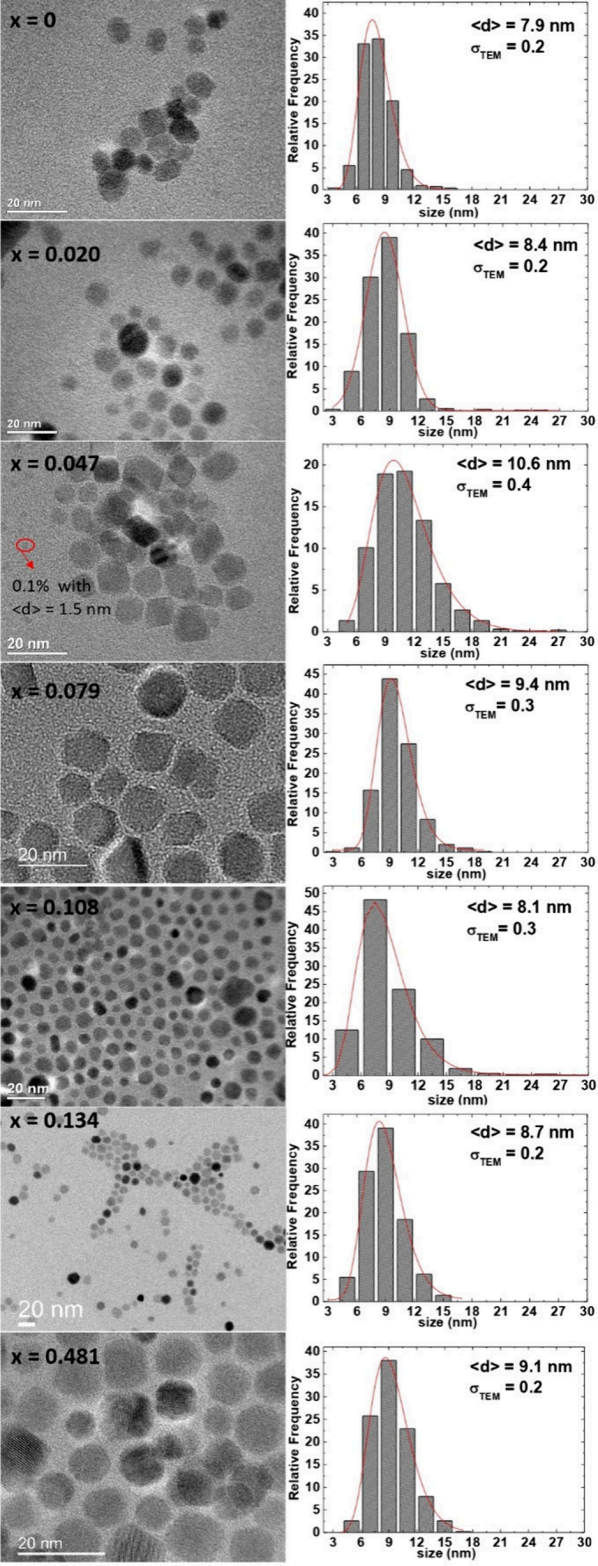
Representative TEM bright field images of Co_*x*_Fe_3–*x*_O_4_ nanoparticles obtained by thermal decomposition method and deposited from their hexane dispersion on an amorphous carbon-coated copper grid and dried at room temperature. The right panel shows the respective diameter histograms fitted with a log-normal distribution, whose parameters are given in the figure and Table 1.

Morphology and size of the magnetic nanoparticles were studied using the TEM, and analysis shows that Co_*x*_Fe_3–*x*_O_4_ MNPs prepared by the thermal decomposition method have good narrow size distribution. Figure 3 shows representative TEM images from Co_*x*_Fe_3–*x*_O_4_ nanoparticles, sizes 7.9–10.6 nm, deposited from their hexane suspension and dried under ambient conditions. The respective diameter histograms are presented in the respective right panels and was built up by considering more than 400 nanoparticles on the analysis. Results were fitted using a log-normal distribution for all samples (presenting a dispersion of at most σ_TEM_ ∼ 0.4). The values of ⟨*d*⟩_TEM_ obtained from the fitting too are given in Table 1. 0.1% of the *x* = 0.047 sample have MNPs of sizes near 1.5 nm; however, the larger ones are the ones that most contribute to the result because of the relative height amount of magnetic moment when compared with the 0.1% of 1.5 nm nanoparticles.

**Figure 4:**
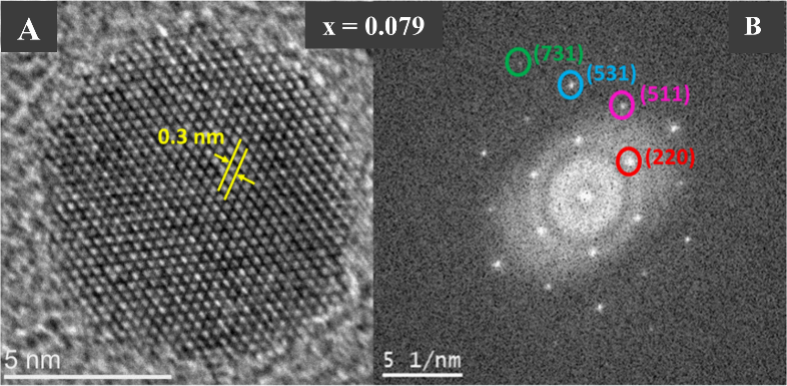
(A) High-resolution TEM image of a single 9.5 nm *x* = 0.079 nanoparticle where the distance of the crystalline planes equivalent to 0.3 nm is seen. (B) Fourier Transform (FFT) of the same nanoparticle; the FFT patterns are indexed with the cubic spinel interplanar distances, magnetite. (Patterns as indicated by the circles of each color.)

Figure 4A shows the structural information on a single nanoparticle from the *x* = 0.079 sample, which was obtained using HRTEM. The distance between the crystalline planes of this nanoparticle is 0.3 nm, corresponding to (220) planes in the spinel-structured magnetite (Fe_3_O_4_). The nanoparticle in this figure is a single crystal, as it corresponds to the group of atomic planes of Fe_3_O_4_. The respective Fourier Transform (FFT) pattern of Figure 4B were obtained by processing image Figure 4A using the Gatan software. The measured lattice spacing based on the rings in the FFT pattern are compared with the known lattice spacing for bulk Fe_3_O_4_, and the found values of [hkl], detailed in the figure, are equivalent to the crystal planes (220), (511), (531), and (731) according to database COD number 1010369.

The ⟨*d*⟩_TEM_ of samples exhibits good agreement with average particle diameter ⟨*d*⟩_XRD_ obtained from XRD (Table 1), indicating that each individual particle is a single crystal. Hence, from both TEM and XRD results, we conclude that the Co_*x*_Fe_3–*x*_O_4_ nanoparticles with uniform size distribution were successfully fabricated using the thermal decomposition method. From now, the grain size ⟨*d*⟩_TEM_ will be used instead of crystal and/or particle size in the following heating and magnetic analysis and discussion.

Also from TEM analyses we corroborate that the morphological characteristics of the nanoparticles remain strongly similar across all samples, despite variations in Co concentration, that implies that the relaxation time and, consequently, SPA will primarily hinge on the modification of *K*_eff_ brought about by the introduction of Co.

**Figure 5:**
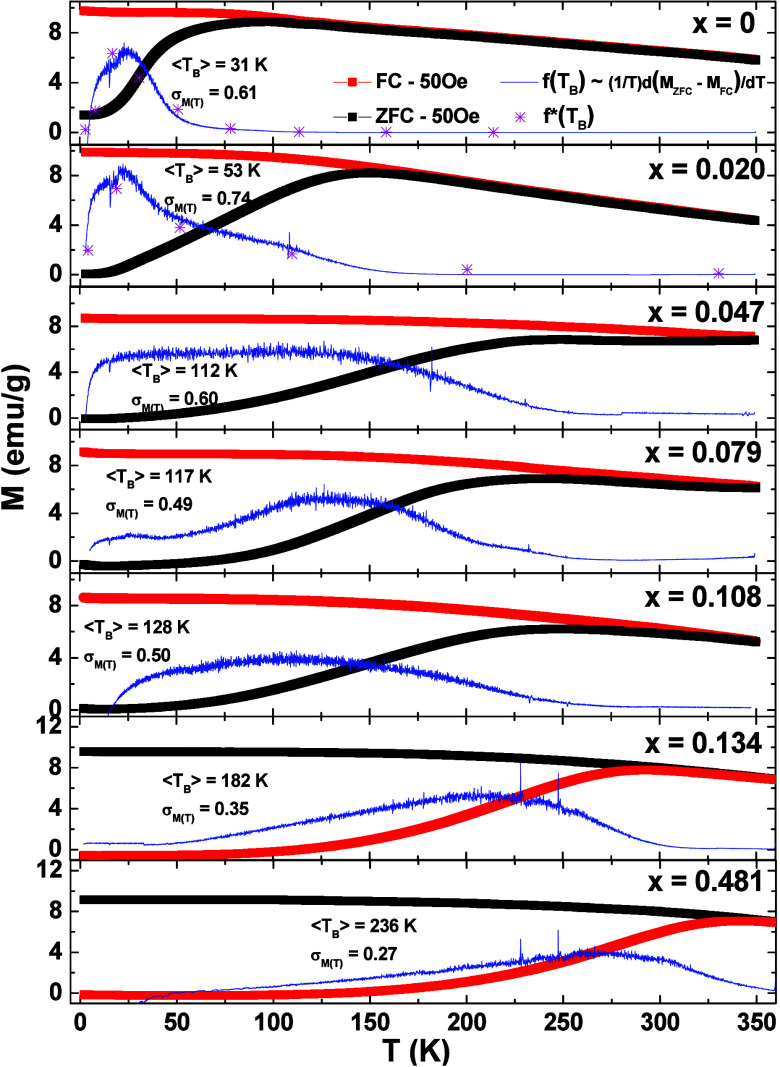
*M*(*T*) all the samples Co_*x*_Fe_3–*x*_O_4_ measurements in ZFC and FC protocols with applied field of *H* = 50 Oe. The blocking temperature distributions *f*(*T*_B_) were calculated for each sample as  (blue line), and f*(*T*_B_) was also calculated using the size distribution of the TEM (pink dotted).

Extensive studies were conducted to determine the magnetic anisotropy of each sample. *M*(*T*) curves were measured using ZFC and FC protocols with a dc field of *H* = 50 Oe, as illustrated in Figure 5. The *M*(*T*) plots offer insights into the distribution of blocking temperatures *f*(*T*_B_), where *T*_B_ represents the temperature distinguishing between superparamagnetic and blocked regimes within a specific measurement time (approximately 100 s in our case). For a noninteracting monodomain magnetic nanoparticles, the *f*(*T*_B_) distribution can be obtained by making^6^. The *f*(*T*_B_) distribution for each sample is illustrated in Figure 5 (blue lines). By computing the weighted average and its standard deviation of *f*(*T*_B_), the ⟨*T*_B_⟩ and the normalized blocking temperature deviation (σ_*T*_B__) was obtained and reported in Figure 5. Utilizing the assumption of a uniaxial anisotropy barrier described by *K*_eff_*V*, the mean blocking temperatures are often expressed as , with *k*_B_ representing the Boltzmann constant.^35^ The ⟨*T*_B_⟩ values obtained from the *M*(*T*) measurements, along with *V* calculated from the average size obtained by TEM, were used to derive the *K*_eff_ for each sample, as presented in Table 2. It is worth mentioning here that a relationship between *K*_eff_ and temperature has previously been established,^36^ wherein our calculations of *K*_eff_ are specifically tied to the average blocking temperature ⟨*T*_B_⟩ of each sample. The most significant expected effect of the incorporation of Co in the samples for this work is the change of the *K*_eff_. It is noteworthy that the obtained σ_*T*_B__ from the *M*(*T*) analysis is nearly three times the values of σ_TEM_ obtained from TEM, as expected, since σ_*T*_B__ is a volumetric quantity and needs to be three times larger than the one-dimensional quantity σ_TEM_. For comparison, f*(*T*_B_) for the sample *x* = 0 and *x* = 0.020 was calculated with the Néel-Brown model (Figure 5, pink dotted) from the size distributions presented in the diameter histogram shown in Figure 3 and assuming a value of τ_0_ = 10^–10^ s, a measurement time *τ*_m_ = 100 s, and using the amplitude and uniaxial energy barrier as fit parameters. The calculations were not carried out for the other samples due to the scarcity of data points in the temperature range from 2 to 350 K; this would require a significantly larger count of particle sizes. These results indicate that, at least for the *x* = 0 and *x* = 0.020 samples, only the size distribution contributes to the *T*_B_ dispersion, and therefore, the compositions of particles are nearly homogeneous.

**Table 2:** Magnetic Parameters Obtained from *M*(*T*) and *M*(*H*) Analysis for All Co_*x*_Fe_3–*x*_O_4_ Samples^a^

Co_*x*_Fe_3–*x*_O_4_ sample name	*K*_eff_ 10^5^ (erg/cm^3^) from *M*(*T*)	σ_*M*(*T*)_	*M*_s_(0) (emu/g)	α	*K*_eff_ 10^5^ (erg/cm^3^) from		*K*_eff_ 10^5^ (erg/cm^3^) from *H*_C_(*T*)	σ_*H*_C__
*x* = 0	4.1	0.61	91.8 ± 0.5	0.29 ± 0.01	1.2 ± 0.1	0.87 ± 0.02	3.1 ± 0.7	0.9 ± 0.2
*x* = 0.020	5.9	0.74	100 ± 1	0.40 ± 0.01	1.6 ± 0.2	0.69 ± 0.02	4.2 ± 0.2	0.9 ± 0.2
*x* = 0.047	6.2	0.60	92.6 ± 0.4	0.69 ± 0.02	4.3 ± 0.3	0.54 ± 0.02	–	–
*x* = 0.079	10.1	0.49	92.5 ± 0.6	0.68 ± 0.02	7.2 ± 0.4	0.45 ± 0.01	–	–
*x* = 0.108	15.9	0.50	94.0 ± 0.5	0.71 ± 0.02	10.0 ± 0.6	0.51 ± 0.01	–	–
*x* = 0.134	18.2	0.35	91.7 ± 0.6	0.78 ± 0.01	12.9 ± 0.5	0.39 ± 0.01	–	–
*x* = 0.481	20.6	0.27	92.3 ± 0.4	0.81 ± 0.02	14.7 ± 0.5	0.33 ± 0.01	–	–

a *K*_eff_ and σ_*M*(*T*)_ is the anisotropy obtained from the *M*(*T*) analysis; *M*_s_(0) is the saturation magnetization at 0 K obtained of the *M*_s_(*T*) analysis; α, *K*_eff_ and  is the anisotropy obtained from the  analysis; and *K*_eff_ and *σ*_*H*_c__ is the anisotropy obtained from the *H*_c_(*T*) analysis.

**Figure 6:**
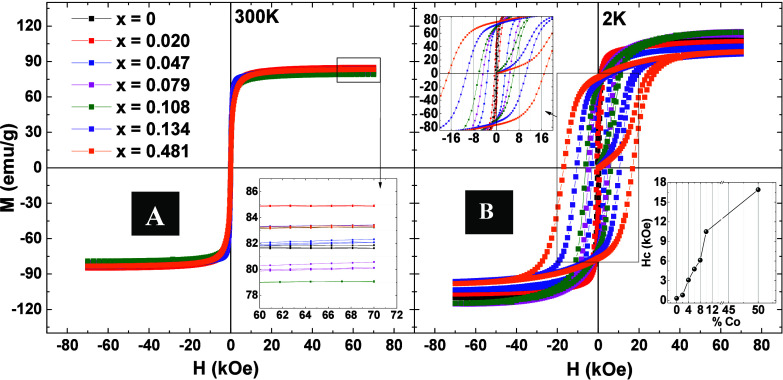
*M*(*H*) all samples Co_*x*_Fe_3–*x*_O_4_ (A) at temperature of 300 K, with the inset showing a zoom in of the *M*_s_, and (B) at temperature of 2 K, with the inset showing a zoom in of the *H*_c_ and the evolution *H*_c_ with the Co content.

The *M*(*H*) at different temperatures was collected in applied magnetization loops with a maximum field of ±70 kOe for all samples. *M*(*H*) at different temperatures, provide information on temperature-dependence of magnetic coercivity (*H*_c_), *M*_s_, and squareness ratio , were *M*_r_ is remanence magnetization at 0 Oe, of the samples. Figure 6A, B shows magnetization loops for all samples at 300 and 2 K respectively. Our results show that *M*_s_ remains almost unchanged at 300 K, with the increase in the Co content in the ferrite, showing very close values and a variation of only ∼6 emu/g between samples. The insets in Figure 6B show the systematic increase of *H*_c_ with increasing Co.^37,38^ This increase is closely related to the change in *K*_eff_,^30^ as will be discussed in more detail below.

**Figure 7:**
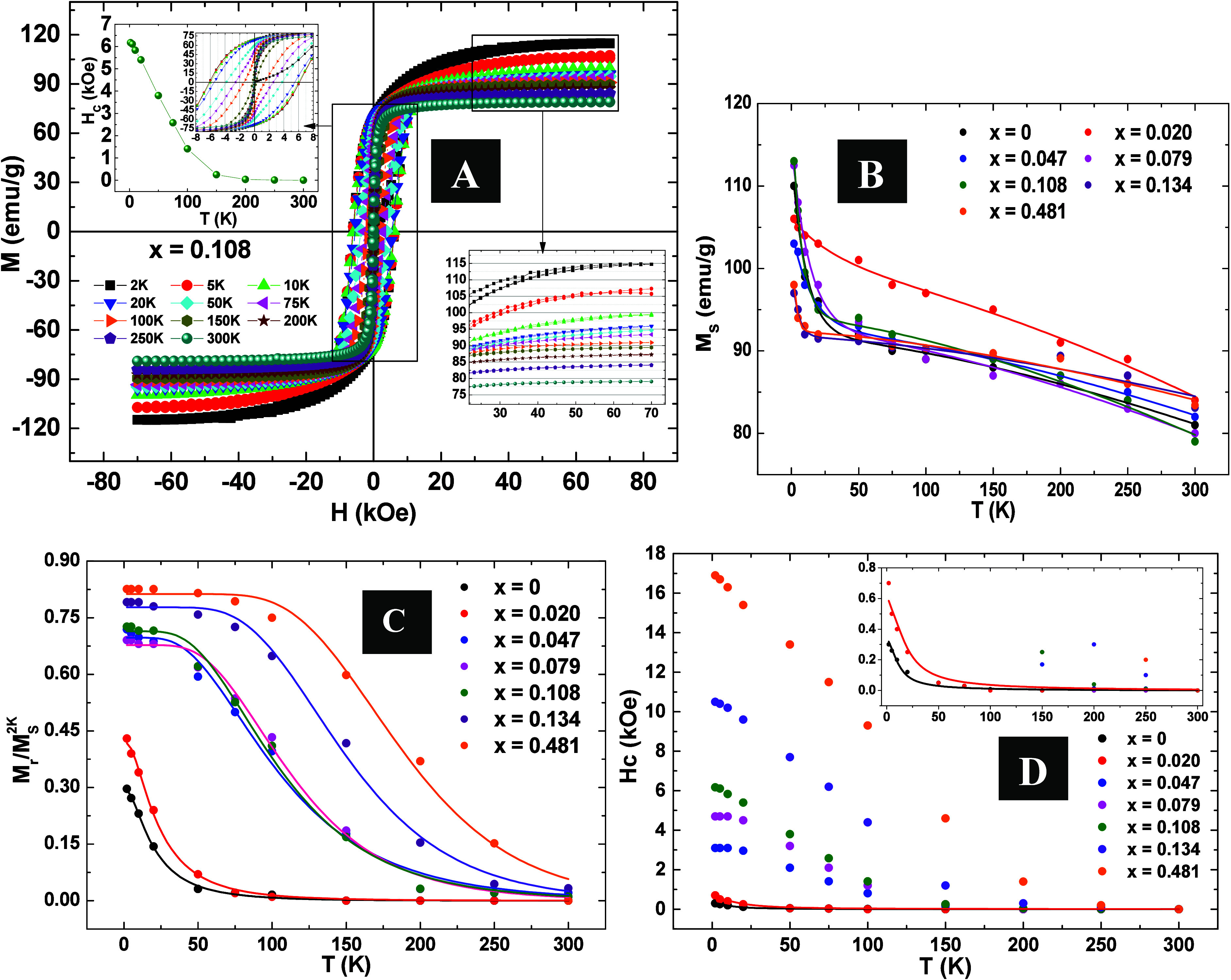
(A) *M*(*H*) for sample *x* = 0.108 at different temperatures, *M*_s_ and *H*_c_ dependence on temperature for this sample is presented in the inset. (B) *M*_s_ dependence on temperature (*M*_s_(*T*)), (C)  dependence on temperature , and (D) *H*_c_ dependence on temperature *H*_c_(*T*) for all the samples Co_*x*_Fe_3–*x*_O_4_ nanoparticles, fitting details, the sample *x* = 0 and *x* = 0.020 is presented in the inset.

As a representation for all samples, Figure 7A shows *M*(*H*) measurements of sample *x* = 0.108 performed at different temperatures and how *H*_c_ and *M*_s_ is strongly temperature dependence. Figure 7B provides a careful analysis of *M*_s_ as a function of temperature (*M*_s_(*T*)) with two distinct dependencies observed: one at high temperatures (above ∼50 K) and another at low temperatures (below ∼50 K). Phenomenological models and experimental studies attributed these two components to the temperature dependence of surface and core magnetization. Previous works have reported an exponential-like decrease in surface spin magnetization with increasing temperature and a Bloch  dependence for core magnetization.^39−41^ Then, the temperature-dependent magnetization, *M*_s_(*T*) can be equated as
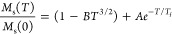
5where *B* represents the Bloch constant, *T*_f_ is the spin freezing temperature, and *A* denotes the surface spin contribution to *M*_s_(*T*). This equation was employed to model the experimental *M*_s_(*T*) curves for all samples. The obtained *M*_s_(0) values, presented in Table 2, align with expectations for Fe_3_O_4_. Notably, *M*_s_(0) exhibited minimal change with varying Co content. All fits yielded similar values for *B* and *A*, where *B̃*(2.5 ± 0.5) × 10^–5^*K*^2/3^ and *Ã*(0.17 ± 0.07). The *T*_f_ values obtained ranged from 3 to 30 K, consistent with findings from previous studies.^39,41^

To obtain insights about the Co effects on magnetic anisotropy and its distribution within the samples, *M*_r_ and *M*_s_ ratios were analyzed (Figure 7C). At 2 K, the  values change from 0.29 to 0.81 depending on Co concentration. The Stoner–Wohlfarth theory predicts, for noninteracting random nanoparticles at 0 K with uniaxial anisotropy, , and for cubic anisotropy, .^42−44^ As the temperature increases, some particles transition to the superparamagnetic state, rendering  for these particles. The temperature-dependence of  is contingent upon the fraction of particles in the blocked state, expressed by
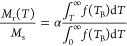
6where . It is worth mentioning that, unlike the *M*(*T*) analyses, the equation above does not assume that the particles present uniaxial anisotropy. On the contrary, information can be obtained about which type of anisotropy are dominant. However, once again, this anisotropy is inherently tied to the temperature ⟨*T*_B_⟩, as it serves as the basis for calculating *K*_eff_. Experimental data for all samples in Figure 7C were fitted using eq 6, and the obtained α is reported in Table 2. The values of α below 0.5 for the *x* = 0 and *x* = 0.020 samples are in concordance with uniaxial anisotropy.^44^ While an α value below 0.5 might seem unconventional, it could be attributed to a number of smaller nanoparticles still unblocked at 2 K, distorting the fitting for these samples.^44^ Conversely, for other samples, as Co concentration increases, α approaches a value near 0.75, indicating a relevant cubic magnetocrystalline anisotropy contribution for these samples.^42−44^ Hence two contributions to the magnetic anisotropy need to be considered: one from magnetocrystalline anisotropy, presenting cubic anisotropy for both Fe_3_O_4_ and CoFe_3_O_4_, and another from surface anisotropy, which is uniaxial and can be significant for small particles like ours. For low Co content, the magnetocrystalline contribution is relatively small, with uniaxial surface anisotropy prevailing. However, as the Co content increases, cubic magnetocrystalline anisotropy takes precedence. The decrease  for each curve with increasing temperature is also related to the reduction of the effective magnetic anisotropy force.^42−44^

Concurrently, the temperature-dependent  allows us to deduce the distribution of blocking temperatures for each sample and, consequently, the effective magnetic anisotropy (*K*_eff_), as listed in Table 2. The *K*_eff_ values obtained from the  analysis are slightly larger than those obtained from the analysis of *M*(*T*) curves; however, both exhibit the same trend with the increase in Co concentration in the samples.

Additionally, *K*_eff_ can be determined by fitting the *H*_c_(*T*) curves, as outlined in the referenced papers.^35,45,46^ According to these works, the temperature dependence of the coercive field can be described by
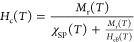
7where *M*_r_(*T*) is given by eq 6,  is the susceptibility of the fraction of superparamagnetic MNPs,  is the coercitive field related only to the blocked NPs at temperature *T* and  is the average blocking temperature, which takes into account only the volume fraction of blocked particles at temperature *T*. This model can describe well experimental *H*_c_(*T*) curves for samples with small amounts of Co; however, it does not fit well for other samples. The curve fittings are shown in Figure 4D for the samples with *x* = 0 and *x* = 0.020, respectively, and the corresponding values of *K*_eff_ and σ_*H*_c__ are presented in Table 2. The obtained values for the anisotropy distribution in this analysis agree with those obtained in the *M*(*T*) analysis for these samples. It is worth mentioning that this model assumes a uniaxial anisotropy for the energy barrier, limiting its applicability to samples with a small amount of Co, specifically, the two samples exhibiting uniaxial anisotropy (*x* = 0 and *x* = 0.020), as depicted in Figure 7D. Moreover, the resultant temperature-dependent *K*_eff_ values are directly linked to the adjusted ⟨*T*_B_⟩, further emphasizing their temperature dependence.

**Figure 8:**
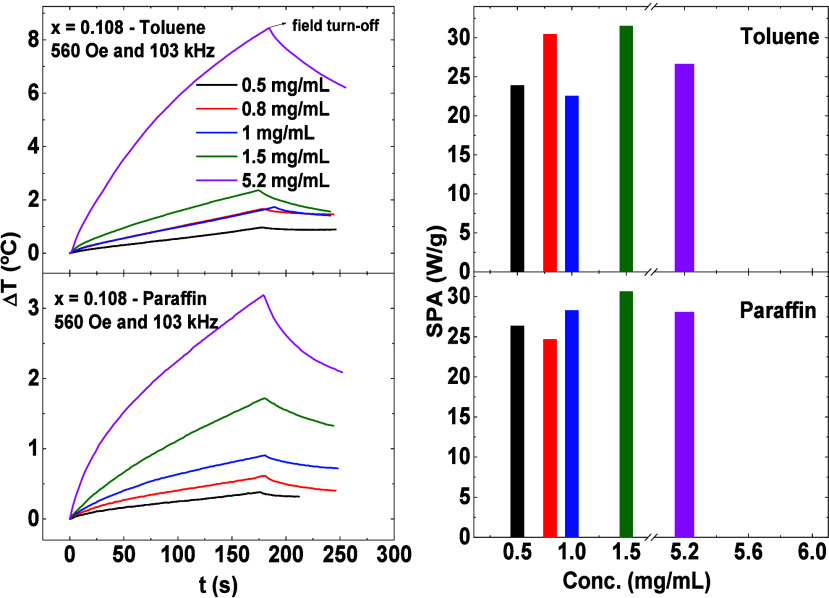
On the left side, MH experiments of magnetic nanoparticles dispersed in toluene (low viscosity) and paraffin (high viscosity) performed in Celes MP 6 kW. The measurements were performed with an ac field of amplitude 560 Oe and frequency 103 kHz for the sample *x* = 0.108 in different concentrations. On the right are their respective values of SPA in toluene and paraffin for each concentration.

Hyperthermia tests were conducted on a magnetic fluid sample (*x* = 0.108) in both toluene (low viscosity) and paraffin (high viscosity) at concentrations ranging from 0.5 to 5.2 mg/mL. The results, depicted in Figure 8, were obtained using an ac field with an amplitude of 560 Oe and a frequency of 103 kHz and performer in Celes MP 6 kW model. The SPA values were determined across different concentrations, ranging between 24 and 31 W/g. These values remain unaffected at varying concentrations and even in the presence of significantly different viscosities in the media, from where can be inferred absence of relevant dipolar interaction among the nanoparticles. Furthermore, Néel relaxation as the primary relaxation mechanism for this sample is evident, for example, in paraffin, where the high viscosity hinders the physical movement of nanoparticles, and the results compared with toluene were equal. As the results do not differ from paraffin to toluene, we concluded that the dominant relaxation mechanism is the Néel for the different samples in toluene and paraffin.

**Figure 9:**
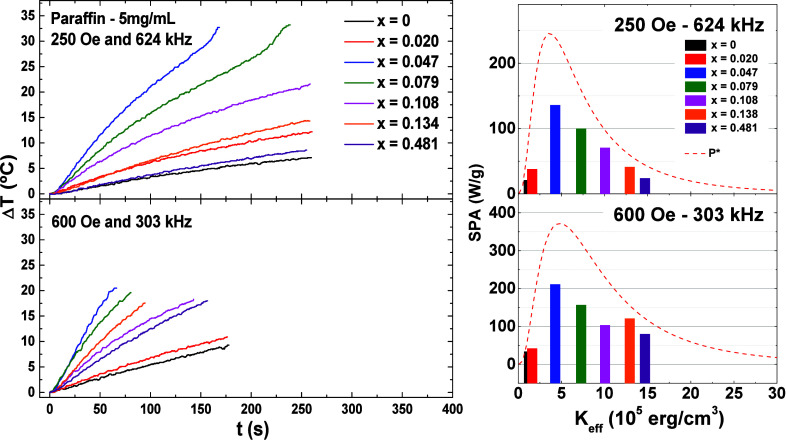
On the left side, representative MH experiments of Co_*x*_Fe_3–*x*_O_4_ MNPs dispersed in paraffin with concentration of 5 mg/mL and performed in the D5 series. On the right are their respective values of SPA as a function of *K*_eff_ obtained from  analysis. The red dashed lines are the calculated power dissipation P*, assuming a size distribution.

A representation of the MH measurements and results of SPA, in paraffin, for all samples are presented in Figure 9. The corresponding SPA values obtained for each sample show a clear dependence on the magnetic anisotropy. Notably, excellent SPA values were achieved, reaching around 200 W/g for the sample with *x* = 0.047 under conditions of 600 Oe and 303 kHz, specifically tailored for particles with an average size of approximately 9 nm.

To validate the obtained SPA values, we estimated the expected SPA by assuming a size distribution and considering only Néel relaxation as the contributing factor. The integration of eq 3 over the size distribution led to the calculated SPA, denoted as P*:
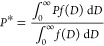
8For this estimation, we employed a log-normal distribution with an average size of 9 nm and a size distribution of 0.27, consistent with the TEM results. Obtained P* values, as a function of *K*_eff_ for the corresponding field amplitudes and frequencies, are depicted as red dashed lines in Figure 9. These theoretical values match *K*_eff_ dependence with the experimental SPA results, affirming that the samples solely undergo Néel relaxation and that we can successfully modulate SPA by altering the magnetic anisotropy, as expected, even knowing that eq 8 is only valid for cases where the anisotropy is uniaxial.

**Figure 10:**
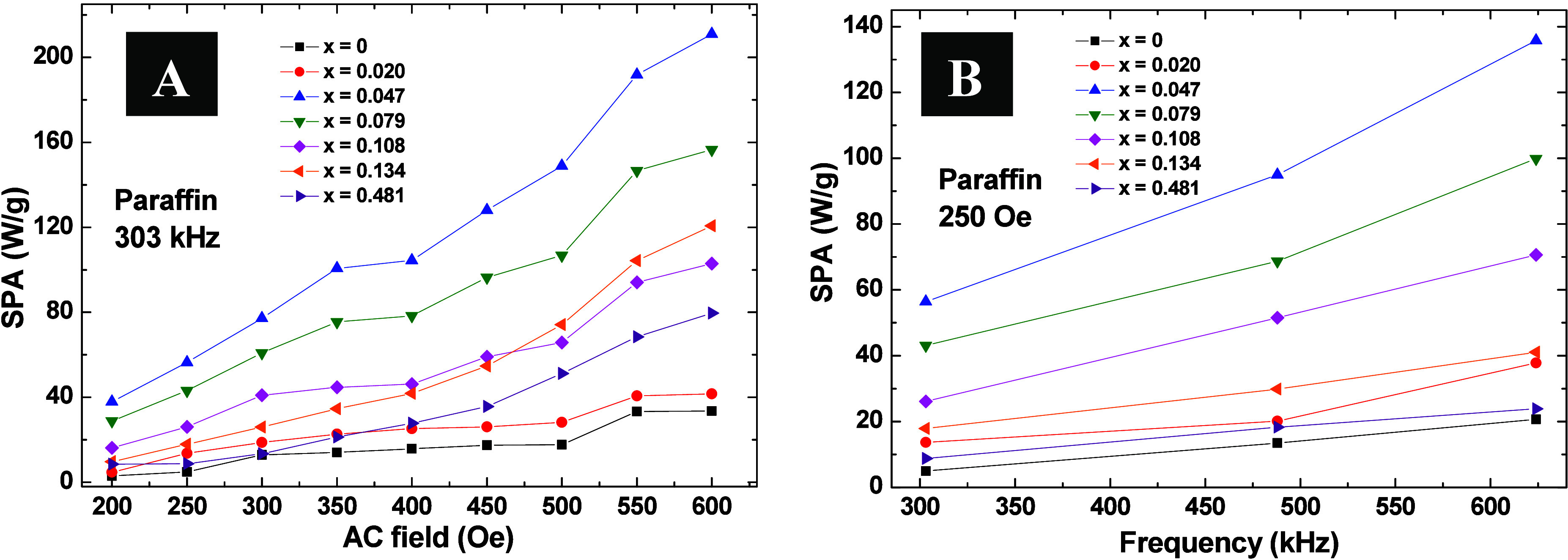
Experimental SPA of Co_*x*_Fe_3–*x*_O_4_ magnetic nanoparticles dispersed in paraffin with a concentration of 5 mg/mL and performed in D5 Series. (A) Obtained SPA for the frequency of 303 kHz as a function of the field amplitude and (B) SPA for a field amplitude of 250 Oe as a function of the frequency.

Figure 10 presents the outcomes of MH experiments for all samples dispersed in paraffin at a concentration of 5 mg/mL. The measurements were conducted across different field amplitudes and frequencies, revealing distinct heating rates attributed to varying effective magnetic anisotropies. For all explored frequencies and ac field amplitudes, the evolution of SPA with Co concentration exhibits a distinct maximum within the concentration range of 0.020 < *x* < 0.079. At the highest frequency of 624 kHz, SPA values escalate from 20.7 W/g (*x* = 0) to 135.7 W/g (*x* = 0.047) before receding to 23.9 W/g (*x* = 0.481). These results underscore the adjustability of *K*_eff_ to optimize SPA values under different experimental conditions.

## Conclusion

In this study, we investigated the effects of composition and magnetism on the magnetic hyperthermia response of Co_*x*_Fe_3–*x*_O_4_ nanoparticles. We have successfully optimized the SPA, by producing MNPs smaller than 10 nm and through the Néel mechanism, for MH applications. Through a detailed exploration of the morphological and magnetic properties of the nanoparticles, we were able to tune the *K*_eff_ by varying the Co content in the samples while keeping the *M*_s_ and ⟨*d*⟩ relatively constant. This study identified an optimal Co concentration (*x* = 0.047) and/or anisotropy (*K*_eff_ = 4.3 × 10^5^ erg/cm^3^) that maximizes SPA (with highest heating efficiency). Furthermore, notably, the substitution of Co led to a transition in the type of *K*_eff_ from uniaxial to cubic, further enhancing our control over the magnetic properties of the nanoparticles.

The identified sweet spot in Co concentration and anisotropy not only enhances SAR but also emphasizes the need for customization in nanoparticle properties to achieve targeted and efficient heat generation across diverse applications. This work significantly contributes to the ongoing endeavor to optimize magnetic nanoparticles for hyperthermia, providing valuable insights into the nuanced adjustments required to elevate heating performance.

Moreover, our observations shed light on the size-dependent Co concentration considerations, elucidating that smaller particle systems necessitate higher Co concentrations for maximizing SPA, while larger particle systems require a smaller amount of Co. This nuanced understanding adds a practical dimension to the broader context of tailoring magnetic properties for enhanced hyperthermia applications. In essence, our study underscores the imperative of fine-tuning magnetic properties and offers a valuable framework for advancing the field of magnetic hyperthermia through targeted nanoparticle design.
